# Next Speakers Plan Their Turn Early and Speak after Turn-Final “Go-Signals”

**DOI:** 10.3389/fpsyg.2017.00393

**Published:** 2017-04-11

**Authors:** Mathias Barthel, Antje S. Meyer, Stephen C. Levinson

**Affiliations:** ^1^Language and Cognition Department, Max Planck Institute for PsycholinguisticsNijmegen, Netherlands; ^2^Psychology of Language Department, Max Planck Institute for PsycholinguisticsNijmegen, Netherlands; ^3^Donders Institute for Brain, Cognition and Behaviour, Radboud UniversityNijmegen, Netherlands

**Keywords:** turn-taking, task-oriented dialogue, production, planning, eye-movements, intonation

## Abstract

In conversation, turn-taking is usually fluid, with next speakers taking their turn right after the end of the previous turn. Most, but not all, previous studies show that next speakers start to plan their turn early, if possible already during the incoming turn. The present study makes use of the list-completion paradigm (Barthel et al., 2016), analyzing speech onset latencies and eye-movements of participants in a task-oriented dialogue with a confederate. The measures are used to disentangle the contributions to the timing of turn-taking of early planning of content on the one hand and initiation of articulation as a reaction to the upcoming turn-end on the other hand. Participants named objects visible on their computer screen in response to utterances that did, or did not, contain lexical and prosodic cues to the end of the incoming turn. In the presence of an early lexical cue, participants showed earlier gaze shifts toward the target objects and responded faster than in its absence, whereas the presence of a late intonational cue only led to faster response times and did not affect the timing of participants' eye movements. The results show that with a combination of eye-movement and turn-transition time measures it is possible to tease apart the effects of early planning and response initiation on turn timing. They are consistent with models of turn-taking that assume that next speakers (a) start planning their response as soon as the incoming turn's message can be understood and (b) monitor the incoming turn for cues to turn-completion so as to initiate their response when turn-transition becomes relevant.

## 1. Introduction

Taking turns at talk in conversation is an essential feature of human interaction. When talking to one another in everyday encounters, interlocutors efficiently align their turns-of-talk, most of the time leaving only very short gaps of about 200 ms (Sacks et al., 1974; de Ruiter et al., 2006; Stivers et al., 2009; Heldner and Edlund, 2010; Levinson, 2016). How they achieve such rapid timing in turn-taking is still largely unresolved (Levinson, 2012). For such neat alignment of talk, next speakers need to (i) start to plan the content of a response to an incoming turn and (ii) recognize the incoming turn's point of completion to know when to launch the articulation of their response. Different turn-taking models have been proposed to explain conversational turn management. They vary in the amount of attention they give to the two tasks faced by next speakers. A group of models developed in the 1970s focuses on the transmission of signals about the state of the current turn at talk (e.g., Duncan, 1972; Duncan and Niederehe, 1974; Duncan and Fiske, 1977). In their approach, the current turn (or speaker) displays signals for turn continuation or yielding which the next speaker could react to when they are displayed. However, most of these cues, prosodic, syntactic, or gestural in nature, are displayed toward the end of the turn, which is arguably too late to start planning a response and initiate articulation without long gaps due to the latencies involved in speech production (Levinson, 2012). Therefore, more recent models of turn-taking formulated the need for early response planning, i.e., preparing the next turn while the incoming turn is still unfolding (Heldner and Edlund, 2010; Levinson and Torreira, 2015)^1^.

In a previous study investigating the timing of task (i), planning of the content of a response, Barthel et al. (2016) came to the conclusion that next speakers begin to plan their response as early as possible, irrespective of how far the current turn's end lies ahead. However, in a dual-task study, Sjerps and Meyer (2015) came to contrary conclusions. In that study, participants tapped their fingers while taking turns with a pre-recorded voice in naming lines of objects on a screen. On the basis of participants' eye-movements and tapping performance, the authors suggested that planning began only at the very end of the incoming turn. The results of a study by Bögels et al. (2015), however, suggested that the participants in their quiz-like experiment started to plan the response to an answer as early as possible, in some cases several words before the end of a question.

Substantial research on task (ii), turn end detection, suggests that, at least in participants overhearing a conversation, projection of the incoming turn's completion point is influenced by the presence or absence of turn-taking cues (Kendon, 1967; Beattie et al., 1982; Schaffer, 1983; Walker and Trimboli, 1984; Cutler and Pearson, 1985; Stephens and Beattie, 1986; Ford et al., 1996; Ford and Thompson, 1996; Caspers, 2003; Wesseling and Son, 2005; Hjalmarsson, 2011). In particular, a study by Lammertink et al. (2015) tested toddler and adult participants while observing a conversation without taking part in it. Both toddlers and adults were found to use both syntactic and intonational cues to turn completion in order to anticipate speaker switches, relying more on syntactic than on intonational cues when these were pitted against each other. Another study by Bögels and Torreira (2015) found that listeners who were asked to press a button upon turn completion take advantage of turn-taking cues that are located close to the turn end. In the corpus that was analyzed to serve as a source of stimuli for that experiment, no early cues to when the turn would end were found. While these studies show that some acoustic cues may be helpful to observers of a conversation, they do not shed light on the question whether interlocutors actually do make use of these cues in conversation. What remains to be shown in order to gain further insight into the organization of human interaction is whether these cues are actually used by speakers to keep gaps between turns short.

The present study was designed to disentangle the relative contribution of early planning on the one hand and reaction to the upcoming turn-end on the other hand to the fast timing of turn-taking that is commonly observed in conversation. It makes use of the list-completion paradigm (Barthel et al., 2016), in which participants listen to sentences of a confederate that contain lists of objects that participants see on a computer screen. The participants' task is to name all objects that are displayed on the screen and have not been named by the confederate. While participants listen to the incoming utterance and eventually prepare and produce their own turn, their eye-movements are tracked as they move their gaze from the objects they need to comprehend to the objects they need to name themselves. The study's design is based on two assumptions: (i) Participants would switch their gaze from confederate objects to participant objects dependent on when they start planning their turn (Griffin and Bock, 2000; Huettig et al., 2011); and (ii) Participants would initiate their response only when they are confident that the incoming turn is complete (Sacks et al., 1974).

Conversation analytic work on German investigated the prosodic tools German speakers have at their disposal to indicate turn-finality vs. turn-continuations. In his work on the functions of intonation for turn-taking in German, Gilles (2005) shows that a falling nuclear contour with a low boundary tone is most widely used in German to mark turn-finality in declarative sentences, whereas rises are used to indicate turn-continuation. This way of marking continuation and termination is very prominent in lists like the ones used in the present experiment, such as *I have a key, a kite, a ruby*. Non-final elements are generally produced with rising pitch, whereas the final element is produced with falling pitch, at least in closed lists, i.e., lists with a finite number of items (von Essen, 1956).

To display that the list under construction is a closed list, speakers can (but need not) use downsteps of successive pitch peaks on list items, which means that the rise in pitch in non-final list elements is lower and lower with every successive element (Féry, 1993; Selting, 2007). These downstepped contours require speakers to pre-plan the length of the list in order to plan the size of the pitch steps. Consequently, listeners could use this early cue to project the length of the list before it comes to be complete.

A third cue to the end of a closed list, next to the two intonational cues, can be a conjunction like *and* that often precedes the final item of closed lists and indicates that the turn will end after the following noun phrase, such as in *I have a key, a kite*, ***and*** *a ruby*. Pitch contours, boundary tones, and lexical cues could therefore be monitored by listeners to identify turn-completion points and used to minimize gaps at turn transitions.

To disentangle the contributions of early planning and reaction to the upcoming turn-end, the present study applied two measures, namely gaze direction as a measure for the timing of planning, and voice onset time as a measure for the latency of launching the response turn. A combination of these two measures can be used to partly disentangle the processes of response preparation and response initiation. Assuming that next speakers aim for short gaps between turns, an earlier start in planning (operationalized as earlier looks for planning in this experiment) should also lead to shorter response latencies (assuming that the head start in planning will not be canceled out by interference of the incoming speech with the planning process). If, however, no difference in planning can be observed in eye-movements, a difference in response latencies should reflect a difference in response initiation. If next speakers can take advantage of any of the cues tested in this experiment to start planning their response early, they should be able to move their gaze for planning earlier and respond faster in turns displaying the cue than in turns without the cue. If however a cue cannot be used to initiate response planning early (e.g., because it was displayed too late in the incoming turn), it could still be useful to detect the end of the incoming turn and to launch the articulation of a response. In that case, the presence of the cue should make no difference to the timing of gaze movements but lead to shorter response latencies compared to the absence of the cue. Early turn-taking cues, including pitch downsteps on non-final items of a list and a lexical cue before the final item of a list, are therefore hypothesized to lead to earlier response planning and consequently shorter gaps between turns. Late cues to turn-completion, however, such as the final boundary tone, were argued to not aid response planning (de Ruiter et al., 2006; Levinson, 2012). Consequently, a turn-final boundary tone can be hypothesized to have no effect on the timing of response planning, but nevertheless it could be useful to detect the turn end and initiate articulation of a response. In that way, it could be used as a “go-signal” for articulation and lead to shorter gaps between turns.

## 2. Materials and methods

The present study uses the list-completion paradigm (Barthel et al., 2016) to investigate the timing of next speakers' response planning and their orientation toward potential cues to turn completion. A confederate talks to a participant and plays pre-recorded critical utterances (recorded by the confederate), so that these utterances seem to be produced live in the flow of conversation. The confederate names the objects visible on her screen and the participant, seeing the same plus a number of further objects, responds what further objects are visible on his or her screen. It can be assumed that participants' gaze follows the objects that are named by the confederate while comprehending the object names, and moves on to the objects that have to be named during response planning (Just and Carpenter, 1980; Griffin and Bock, 2000; Tanenhaus et al., 2000; Griffin, 2001; Altmann and Kamide, 2007; Huettig et al., 2011). The experiment was conducted in German and the critical utterances of the confederate appeared in the following conditions, exemplified in (1) to (4).

1. *Ich habe einen Schlüssel, einen Lenkdrachen, einen Rubin*. (L%)
  I have a key, a kite, a ruby.
2. *Ich habe einen Schlüssel, einen Lenkdrachen **und** einen Rubin*. (L%)
  I have a key, a kite and a ruby.
3. *Ich habe einen Schlüssel, einen Lenkdrachen, einen Rubin*. (**M%**)
  I have a key, a kite, a ruby.
4. *Ich habe einen Schlüssel*, einen Lenkdrachen, *einen Rubin*. (**DWNS**, L%)
  I have a key, a kite, a ruby.

**Figure 1:**
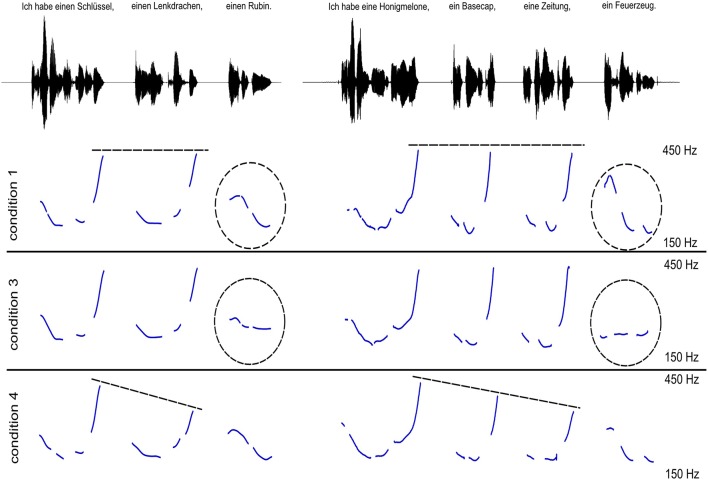
**Two examples illustrating the intonation contours used in conditions 1 (baseline condition), 3 (no boundary tone condition), and 4 (downstepped condition)**. Condition 1 (and equally condition 2, not displayed here) contains no downsteps on non-final list items and a low boundary tone at the turn end. By contrast, condition 3 contains no final low boundary tone. Condition 4 contains downsteps and a final low boundary tone.

Sentences in condition (1) (baseline condition) did not contain a lexical cue (like *and*) to mark the final item of the list (−LEX) and ended in a low falling boundary tone (+BT). Non-final list items were produced with high rising intonation and pitch peaks of equal height around 400 Hz, i.e., without downsteps of pitch peaks on non-final list items (−DWNS). Sentences in condition (2) (lexical cue condition) were similar to sentences in condition (1), except that the lexical cue *und* (“and”) preceded the final list item to mark the item as being the last one of the list (+LEX; if the sentence contained only one item, *nur* “only” was used instead of “and”). Sentences in condition (3) (no boundary tone condition) were the same as in condition (1), except that their final intonation contour was manipulated to end in a flat mid tone instead of a low falling boundary tone (−BT). Sentences in condition (4) (downstepped condition) were similar to condition (1), except that non-final list items were produced with consecutive downsteps in pitch peaks in non-final list items (+DWNS). Figure 1 shows the difference in intonation contours between sentences in conditions (1), (3), and (4).

### 2.1. Participants

Thirty-eight German native speakers (mean age = 22.8 years; *SD* = 2.9) were tested as paid participants at the MPI for Psycholinguistics. All participants reported normal or corrected-to-normal vision and normal hearing abilities. Data of three participants were not considered in the analyses due to technical failure during recording. Of the remaining participants, 10 answered “yes” to a post-experiment query whether pre-recorded materials were presented to them during the experiment. This factor was included as a binary control variable in the analyses (±recording_noticed). The experiment was approved by the Ethics Committee of the Faculty of Social Sciences, Radboud University Nijmegen. Written informed consent was obtained from all subjects.

### 2.2. Apparatus

The participant and the confederate were seated in separate cabins in front of and about 60 cm away from 21 inch computer screens. They were unable to see each other and could only communicate via microphones and headphones. The participants' eye-movements were recorded with an SMI RED-m remote eye-tracker (120 Hz).

### 2.3. Visual stimuli

Four-hundred and twenty-four pictures of concrete objects that were used in the study by Barthel et al. (2016) were used in the experiment. All pictures, with the exception of twenty pictures used in practice trials, showed inanimate objects.

**Figure 2:**
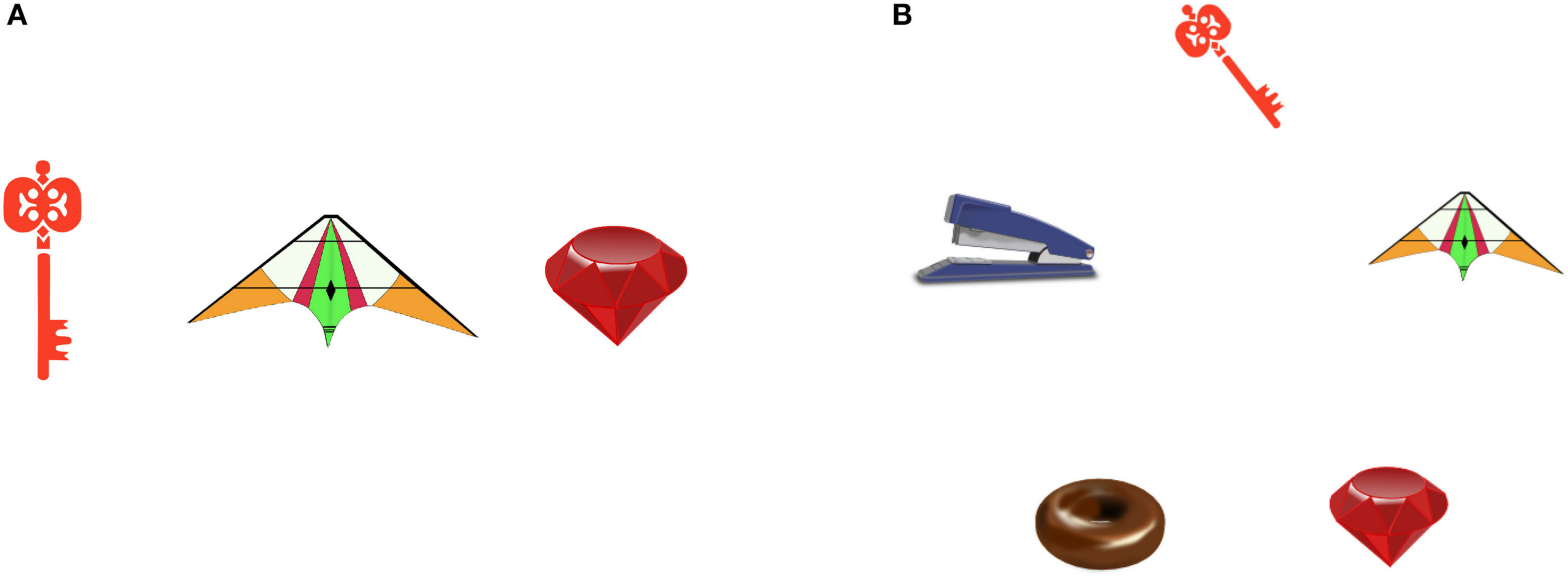
**Example item displays**. **(A)** Confederate display. **(B)** Participant display. Reproduced from Barthel et al. (2016).

One-hundred and four pairs of displays (participant displays and corresponding confederate displays) that showed a differing number of objects drawn from the pool of object pictures were used as visual stimuli (see Figure 2 for an example). The participant displays showed between three and five objects, including all objects shown on the corresponding confederate display plus zero, one, two, or three further objects. In participant displays that showed three objects, the objects formed an equilateral triangle, when showing four objects, the objects formed a square, when showing five objects, the objects formed an equilateral pentagon.

Ninety-two displays were critical test displays, with twenty-eight displays each showing three, twenty-eight showing four, and thirty-six showing five objects on the participant display. The confederate displays showed between zero and five objects, so that twelve participant displays showed no more objects than the corresponding confederate display; twenty-eight participant displays showed one more object, twenty-eight participant displays showed two more objects, and twenty-four participant displays showed three more objects. The experiment was preceded by a practice phase using twelve display pairs, with four participant displays each showing three, four, or five objects.

### 2.4. Auditory stimuli

Sentences accompanying the visual displays were pre-recorded, using a unidirectional Sennheiser ME64 microphone attached to a digital flash recorder. Each sentence was recorded in conditions 1, 2, and 4. Sentences in condition 3 (no boundary tone condition) were manipulations of the corresponding sentences in condition 1 (baseline condition). The final low falling boundary tone was flattened to a mid level with Praat (Boersma and Weenink, 2015). Sentences in condition 4 (downstepped condition) were recorded and selected to contain downsteps of pitch peaks on non-final list items, with the first item peaking at about 400 Hz and the penultimate item peaking at about 340 Hz. The more items a list contained, the smaller were the differences in pitch peaks between adjacent list items. Sentences that contained 3 list items were produced with a downstep of 50–70 Hz. Sentences that contained 4 list items were produced with two downsteps of 30–40 Hz. Sentences that contained 5 list items were produced with three downsteps of 15–40 Hz. The pauses between object nouns were manipulated with Praat to have a random length between 400 and 600 ms, equal for the different versions of each sentence, imitating the average length in the original recordings.

Eight sentences did not contain any object nouns and were used as fillers. In these sentences, the object list was replaced by *nichts* (“nothing”), as in *Ich habe nichts* (“I have nothing”). Sentences accompanying the twelve practice trials were produced live. These sentences were produced to sound similar to the pre-recorded sentences, using the formats that were otherwise used in conditions 1, 2 and 4 in the pre-recorded sentences.

### 2.5. Items and design

A participant display in combination with the accompanying sentence constituted an experimental item. In sixty-one of the items in which the confederate named at least one object, the objects were arranged in clockwise order as they were named, starting at the top of the display. In twenty-three of the items, other arrangements were used, so that the participants had to listen attentively and search for the items mentioned by the participant, rather than scanning the objects in the same order on all trials.

Four lists were constructed, with each sentence and the accompanying display appearing once per list. Since sentences with less than three objects could not appear in condition 4 (downstepped condition), and sentences with less than two objects could not appear in condition 3 (no boundary tone condition), the number of items per condition was not balanced throughout the experiment. In each list, twenty-eight items appeared in condition 1, twenty-eight items in condition 2, sixteen items in condition 3, and twelve items in condition 4. Each participant was assigned to one of the lists.

### 2.6. Procedure

#### 2.6.1. Familiarization and instructions

The procedure followed Barthel et al. (2016). Participants were invited to the lab to take part in a dialogue experiment. Upon arrival, they were given a picture booklet containing all pictures used in the experiment and asked to name them. In case a participant could not recognize or name a picture, a name was provided by the experimenter. The experimenter noted down participants' responses. The familiarization phase was audio-recorded.

After the familiarization phase, the confederate arrived and was introduced as a second participant. Participant and confederate were informed that they would be seated in separate cabins and talk to each other via headphones and microphones to play the following game. They would see a number of displays on their respective screens, showing a number of objects. All objects that were displayed on the confederate display were also displayed on the participant display. The confederate was to tell the participant which things she has got on her display, so that the participant could tell the confederate what *further* objects (s)he has got. Participants were not instructed to use any particular utterance format.

The confederate was instructed to try to remember which objects she had seen and which names she had heard. This served as a cover task to distract participants from the aim of the study. Participants were told that their eye-movements would be recorded in order to study looking behavior when searching for objects on a screen whose names were heard. After instructions were given, the eye-tracker was calibrated and calibration was repeated three times during the experiment.

### 2.7. Test phase

Before the test phase, participants completed twelve practice trials. During the test phase, all communication between the participants and the confederate was live, except for the critical pre-recorded sentences. The confederate started the trials and the corresponding pre-recorded utterances so as to make them fit naturally into the conversation.

Participants were asked to look at a fixation cross that was presented in the center of the display at the beginning of each trial, which triggered the presentation of the item displays. After a preview that varied randomly between items between 600 and 1,000 ms, the stimulus sentence began.

After the experiment, participants were asked in a computerized questionnaire whether they had noticed the presence of pre-recorded speech. The answers were used as a control variable (±recording_noticed).

The experiment took about 25 min. The entire test session took about 1 h, including familiarization, test, and questionnaire.

## 3. Results

Statistical analyses were based on linear mixed effects regression models fitted in R (R Core Team, 2014) using the package lme4 (Bates et al., 2014). Participants' fixation preferences and response latencies were the dependent variables. The maximal random effects structure justified by design was used for all models (Barr, 2013; Barr et al., 2013). Control variables were not included in the random effects structure. All categorical variables were dummy coded (0 and 1). Statistical significance was assessed with *F*-tests with Kenward-Roger approximations of degrees of freedom (Kenward and Roger, 1997; Fox and Weisberg, 2011; Halekoh and Højsgaard, 2014).

### 3.1. Response timing

Response latencies for critical turn transitions were measured manually with Praat (Boersma and Weenink, 2015). They were coded as time intervals between the end of the incoming turn and the beginning of the response turn, excluding any non-speech sounds like audible in-breaths. Participants always named the correct objects that were not named by the confederate. Response latencies ranged from −56 ms (short overlap) to 5,113 ms (*M* = 1,002 ms, *SD* = 432 ms, *N* = 3,220). The present latencies are relatively long compared to averages observed in natural conversation, probably due to task demands. They are comparable to the latencies obtained by Barthel et al. (2016), who used the same paradigm. Table 1 shows an overview per condition. For the statistical analyses, thirty-four data points (1%) were removed from the data set since they were outliers of more than three standard deviations of the mean response latency of the respective subject that produced the data-point.

**Table 1:** **Response latencies by condition**.

**Condition**				**Mean (SE)**	***N***
**Number**	**LEX**	**BT**	**DWNS**		
1	−	+	−	1,010 (12)	988
2	+	+	−	922 (12)	990
3	−	−	−	1077 (14)	560
4	−	+	+	873 (18)	402

*Means and standard errors (SE) in ms*.

Since confederate turns in the different conditions differ in their average number of objects that are named by the confederate, they are inherently of different average lengths. Because of this difference, the duration of the critical turns was included as a control variable in the analysis.

To test for the effects of interest, a model was designed to fit response latencies. It included presence of lexical cue (±LEX), presence of low falling boundary tone (±BT), and presence of downstepped pitch peaks (±DWNS) as predictors and the duration of the confederate turns in seconds, as well as ±recording_noticed as control variables. Response latencies were significantly longer in −LEX items (condition 2) than in the baseline condition [β = 90, *SE* = 19, *F*_(1, 35)_ = 21.04, *p* < 0.001], i.e., participants responded slower when no lexical cue to the turn end was present. Furthermore, response latencies were significantly longer in −BT items (condition 3) than in the baseline condition [β = 60, *SE* = 22, *F*_(1, 34)_ = 7.39, *p* = 0.01], i.e., participants responded slower when no final intonational cue to the turn end was present. ±DWNS did not significantly influence response latencies, meaning that the apparent difference in the descriptive statistics is merely an artifact of sentence duration^2^. Duration of the confederate turn had a significant effect on response latencies [β = −49, *SE* = 7, *F*_(1, 85)_ = 42.95, *p* < 0.001], meaning that participants responded faster, the longer the incoming turn, presumably because participants' level of preparedness to speak increases as the likelihood that the incoming turn will come to an end increases (cf. Magyari et al., 2017). Table 2 shows a model summary.

**Table 2:** **Response timing model and *F*-tests**.

	**Estimate**	***SE***	***t***	***F***	**Sig**.
(Intercept)	953.482	53.4	17.830		
lexical cue_no	90.190	19.6	4.602	*F*_(1, 35)_ = 21.041	***
boundary tone cue_no	60.344	22.0	2.741	*F*_(1, 34)_ = 7.391	**
downsteps_no	8.663	34.4	0.252	*F*_(1, 35)_ = 0.061	n.s.
sentence_duration	−48.974	7.1	−6.836	*F*_(1, 85)_ = 42.957	***
recording_noticed_yes	74.624	73.087	1.021	*F*_(1, 32)_ = 0.878	n.s.

Formula: RT ~ 1 + LEX + BT + DWNS + recording_noticed + sentence_duration_centered + (1 + LEX + BT + DWNS | subject) + (1 + LEX + BT + DWNS | item). Presences of cues were used as reference levels, so that effects shown are effects of absence of cues. Asterisks indicate significance levels of effects. *p < 0.05;

** p = 0.01;

*** *p < 0.001*.

### 3.2. Eye-movements

**Figure 3:**
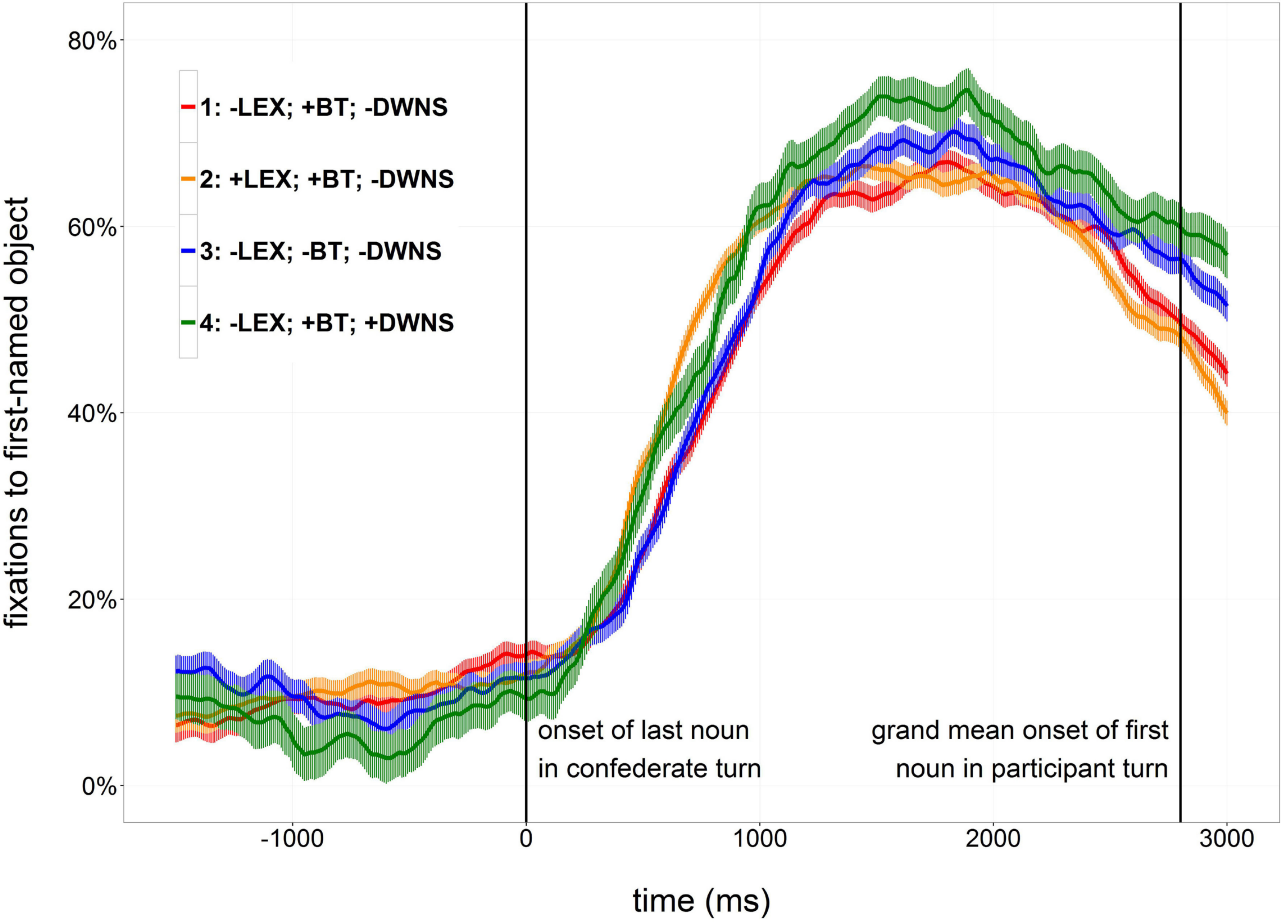
**Proportions and standard errors of looks to the target object time-locked to the onset of the last object noun of the confederate turn (0 ms)**.

In order to investigate the time course of participants' planning of their response to critical confederate turns, fixations to the first-mentioned objects in the participants' responses (target objects) were analyzed. Fixations toward an area of interest covering the target objects and approximately 0.25 degrees of visual angle around them were categorized as target fixations. Figure 3 shows proportions of target fixations time-locked to the beginning of the last object noun in the confederates utterance.

Participants' eye-movements were analyzed in a time window from 0 ms until 2,600 ms, corresponding to the beginning of the last noun in the confederate's turn (0 ms) and the grand mean duration from the time-lock point until the beginning of the first object noun in the participant turn (2,600 ms), respectively. Fixations to the target object were aggregated to empirical logits in 100 ms time bins over the course of the analysis window by subjects and by items, respectively (Barr, 2008). The empirical logit transformation removes statistical dependencies in the data, which is important to satisfy the assumptions of linear regression. Only trials that included both looks for production and looks for comprehension were analyzed, excluding trials in which the confederate named none or all of the displayed objects. Seventy-eight of the remaining trials were discarded due to trackloss, i.e., missing data for a consecutive stretch longer than 500 ms within the time window of analysis. The final data set included 2,442 trials.

Eye-movement patterns were analyzed using hierarchical quasi-logistic growth curve modeling (Mirman, 2014). Growth curve analysis is a variety of mixed effects regression that uses orthogonal polynomial time terms as predictors to model differences in curve shapes, in this case differences in growths of fixation likelihoods as expressed by empirical logit transforms. Linear, quadratic, and cubic orthogonal time terms were included as predictors in the model. The linear time term (Time) models the overall increase in fixations over the time course of a trial. The quadratic time term (Time^2^) models the steepness of the curve, i.e., how “U-shaped” it is. The cubic time term (Time^3^) describes at what point in time fixations increase (“S-shaped” curve). An interaction of the linear time term with a factor of interest would signify a difference in the slope of the increase of proportions over time in one level of the factor vs. another level. An interaction of the quadratic time term with a factor of interest would signify a difference in the speed with which proportions increase in one level of the factor vs. another level, thereby describing the pointedness of a U-shaped curve. An interaction of the cubic time term with a factor of interest would signify a difference in latency, i.e., a difference in when proportions start to increase in one level of the factor vs. another level. This interaction is most interesting to us, since it models the predictions about when participants shift their gaze toward the target objects in the different conditions. Table 3 shows an overview of model summaries and significance levels.

**Table 3:** **Eye-movement results of by-subject analysis**.

**Comparison**	**Effect**	**β**	***SE***	***F***	**Sig**.
Cond. 1 vs. cond. 2 (±LEX)	t^1^ × cond.	3.30	0.29	*F*_(1, 345)_ = 6.51	*
	t^2^ × cond.	−2.98	0.21	*F*_(1, 740)_ = 4.03	*
	t^3^ × cond.	−0.51	0.19	*F*_(1, 1116)_ = 6.21	*
Cond. 1 vs. cond. 3 (±BT)	t^1^ × cond.	0.47	0.34	*F*_(1, 270)_ = 1.64	n.s.
	t^2^ × cond.	−0.52	0.29	*F*_(1, 255)_ = 2.90	n.s.
	t^3^ × cond.	0.17	0.19	*F*_(1, 924)_ = 0.76	n.s.
Cond. 1 vs. cond. 3 (±DWNS)	t^1^ × cond.	−0.25	0.35	*F*_(1, 161)_ = 0.43	n.s.
	t^2^ × cond.	−0.24	0.34	*F*_(1, 181)_ = 0.46	n.s.
	t^3^ × cond.	0.23	0.26	*F*_(1, 273)_ = 0.71	n.s.

Formula = emplogit ~ (time1 + time2 + time3) * condition + (1 + (time1 + time2 + time3) * condition | subject/item) t^2^ = TIME^2^, t^3^ = TIME^3^. β's indicate effects of absence of cues. Asterisks indicate significance levels of effects.

* p < 0.05;

**p < 0.01; ***p < 0.001. By-item analysis yielded a similar pattern of results.

Visual inspection of the proportions of fixations indicates that proportions of target looks are generally at a low level during the incoming turn, increase suddenly after the onset of the last noun of the confederate turn and start to decrease again after about two seconds (Figure 3). In condition 2, which contains a lexical cue to the turn end, the initial increase in proportions of target looks is steeper and takes place earlier than in condition 1, which does not contain this cue. Similarly, the initial increase in proportions in condition 4, containing downsteps of pitch peaks in non-final list items, seems to be slightly steeper than in condition 1, not containing downsteps. Proportions of target looks in condition 3, containing no low falling boundary tone, and in condition 1 do not obviously differ.

Conditions 1 and 2 were compared to test for effects of the lexical cue to turn end (±LEX). Both by-subject and by-item comparisons showed interaction effects of Time^2^× LEX and Time^3^× LEX in the direction of earlier and steeper increases in trials with a lexical cue than in trials without a lexical cue.

Conditions 1 and 3 were compared to test for effects of a boundary tone cue (±BT). No interaction of Time^2^× BT or Time^3^× BT was found to be significant, indicating that proportions of target looks were not modulated by the presence or absence of a final low boundary tone.

Conditions 1 and 4 were compared to test for effects of downsteps in pitch peaks on non-final list items (±DWNS). No interaction of Time^2^ × DWNS or Time^3^ × DWNS was found to be significant, indicating that the presence or absence of downstepped pitch peaks on non-final list items had no influence on the growth of proportions of target looks.

## 4. Discussion

The present study set out to investigate whether, in a conversation, next speakers make use of cues to turn ends to align the beginning of their next turns to the end of the incoming turn. Three types of cues were tested: a lexical cue that indicated that the turn would end after the following noun phrase, a final boundary tone that prosodically marked the turn as complete, and a pitch contour that allowed for an early estimation of the length of the unfolding turn. To test the use of these different cues in turn-taking, an experiment using the list-completion paradigm was designed, in which a naive subject and a confederate took turns in naming objects (Barthel et al., 2016). Which objects participants had to name depended on the objects that were named in the critical turns by the confederate. These critical turns either did or did not contain the relevant turn taking cues. The conversation of participant and confederate and the participant's eye-movements were recorded to analyze at what moment participants planned and initiated their response turns.

Participants were found to start planning their turn as soon as they knew which objects they had to name, replicating the results of Barthel et al. (2016). When the lexical cue *und* (“and”) was present before the last item of the list of the incoming turn, participants knew the following list item to be the last item before the turn would be complete. In sentences containing this lexical cue, participants started planning their response earlier than in sentences not containing this cue, showing that they started planning their response as soon as possible. Dependent on when the turn-final boundary tone becomes recognizable (also indicating the end of the turn) the lexical cue gave participants a head-start in response planning of at least the length of one syllable^3^. Through this head-start in response planning, participants could respond faster after turns with a lexical cue to the turn end than after turns in the baseline condition.

Contrary to the lexical cue, which was located before the last noun phrase of the turn, the final boundary tone was located right before the end of the turn. It was argued before that turn-taking cues which are located at the end of a turn could not be used to time the planning of the content of the response (de Ruiter et al., 2006; Levinson, 2012). The present study supports this argument. No difference in the timing of looks for response planning was found between turns that did contain a turn-final prosodic cue and turns that did not. However, participants were found to rely on turn-final cues to minimize the gap between turns. Response times were faster after turns containing a turn-final boundary tone than after turns not containing this cue. This pattern of results suggests that turn-final cues to the turn-end are irrelevant for the timing of response planning but help next speakers to time the initiation of their turn. Consequently, next speakers seem to use turn-final cues as “go-signals” to launch their response when turn transition becomes relevant. The combination of the absence of an effect on the timing of response planning as measured by participants' gaze movements and the presence of an effect on their response latencies shows that the combination of these two measures makes it possible to differentiate between the two processes, response preparation and response initiation.

No evidence was found that next speakers make use of the early prosodic downstep cue to turn length. Participants were not found to use downsteps on pitch peaks in list items to plan their response earlier or respond faster than in turns without a downstepped pitch contour. This early prosodic cue could have been used by participants as much as the lexical cue to the last list item, since the number of list items might have been guessed from the size of the downsteps. However, it is less discrete than the lexical cue, which might be the reason why participants relied on this cue less than on the lexical cue. Both findings on the use of prosodic cues are in line with the conclusions drawn by Bögels and Torreira (2015), who found that participants in their experiments only relied on final intonational turn-taking cues but not on turn-initial intonational cues when trying to detect turn-completion points.

In conclusion, the results suggest that next speakers plan the content of a response as early as the incoming turn's message becomes recognizable and that turn-final cues can function as “go-signals” to initiate response in a timely fashion. Given that lists are a natural kind of conversational turn that is frequently encountered in everyday situations, the present results can be assumed to be generalizable to casual conversation (Selting, 2007). Turn-final cues can therefore be assumed to be used by speakers to indicate turn-yielding and next speakers can orient to them so as to minimize gaps when taking the floor. The findings show that response turn preparation and the timing of its articulation need to be regarded as separate processes. Response planning depends on (an anticipation of) the incoming turn's message, while response initiation depends on the next speaker's confidence that the incoming turn comes to conclusion and that speaker transition becomes relevant. Consequently, the findings support turn-taking models that include early content planning and the use of turn-final cues as “go-signals” to initiate response (e.g., Sacks et al., 1974; Levinson and Torreira, 2015).

## Author contributions

MB designed and ran the experiment and analyzed the data. MB wrote the paper and AM and SL commented on it.

## Funding

This research was financed by the ERC Advanced grant # 269484 INTERACT awarded to SL and by the Max Planck Institute for Psycholinguistics.

### Conflict of interest statement

The authors declare that the research was conducted in the absence of any commercial or financial relationships that could be construed as a potential conflict of interest.

## Footnotes

^1^The idea of turn prediction and early planning was already present in the simplest systematics of turn-taking by Sacks et al. (1974).

^2^A separate model was run to test for the effect of ±DWNS in the subset of items that are directly comparable to one another, i.e., that have at least three confederate objects plus at lease one additional participant object. The pattern of results is the same as in the full model, showing no effect of ±DWNS.

^3^The average length of the lists' final nouns was 670 ms.

## References

[B1] Altmann G. T. Kamide Y. (2007). The real-time mediation of visual attention by language and world knowledge: linking anticipatory (and other) eye movements to linguistic processing. J. Mem. Lang. 57, 502–518. 10.1016/j.jml.2006.12.004

[B2] Barr D. J. (2008). Analyzing visual world eyetracking data using multilevel logistic regression. J. Mem. Lang. 59, 457–474. 10.1016/j.jml.2007.09.002

[B3] Barr D. J. (2013). Random effects structure for testing interactions in linear mixed-effects models. Front. Psychol. 4:328. 10.3389/fpsyg.2013.00328 23761778 PMC3672519

[B4] Barr D. J. Levy R. Scheepers C. Tily H. J. (2013). Random effects structure for confirmatory hypothesis testing: keep it maximal. J. Mem. Lang. 68, 255–278. 10.1016/j.jml.2012.11.001 24403724 PMC3881361

[B5] Barthel M. Sauppe S. Levinson S. C. Meyer A. S. (2016). The timing of utterance planning in task-oriented dialogue: evidence from a novel list-completion paradigm. Front. Psychol. 7:1858. 10.3389/fpsyg.2016.01858 27990127 PMC5131015

[B6] Bates D. Maechler M. Bolker B. Walker S. (2014). Fitting Linear Mixed-Effects Models Using lme4. J. Stat. Softw. 67, 1–48. 10.18637/jss.v067.i01

[B7] Beattie G. W. Cutler A. Pearson M. (1982). Why is Mrs Thatcher interrupted so often? Nature 300, 744–747. 10.1038/300744a0

[B8] Boersma P. Weenink D. (2015). Praat: Doing Phonetics by Computer [Computer Program]. Version 5.3.56. Available online at: http://www.praat.org

[B9] Bögels S. Magyari L. Levinson S. C. (2015). Neural signatures of response planning occur midway through an incoming question in conversation. Sci. Rep. 5, 1–11. 10.1038/srep12881 26242909 PMC4525376

[B10] Bögels S. Torreira F. (2015). Listeners use intonational phrase boundaries to project turn ends in spoken interaction. J. Phonet. 52, 46–57. 10.1016/j.wocn.2015.04.004

[B11] Caspers J. (2003). Local speech melody as a limiting factor in the turn-taking system in Dutch. J. Phonet. 31, 251–276. 10.1016/S0095-4470(03)00007-X

[B12] Cutler A. Pearson M. (1985). On the analysis of prosodic turn-taking cues, in Intonation in Discourse, ed Johns-Lewis C. (London: Croom Helm), 139–155.

[B13] de Ruiter J. Mitterer H. Enfield N. (2006). Projecting the end of a speaker's turn: a cognitive cornerstone of conversation. Language 82, 515–535. 10.1353/lan.2006.0130

[B14] Duncan S. (1972). Some signals and rules for taking speaking turns in conversations. J. Pers. Soc. Psychol. 23, 283–292. 10.1037/h0033031

[B15] Duncan S. Fiske D. W. (1977). Face-to-Face Interaction: Research, Methods, and Theory. Hillsdale: Lawrence Erlbaum.

[B16] Duncan S. Niederehe G. (1974). On signalling that it's your turn to speak. J. Exp. Soc. Psychol. 10, 234–247. 10.1016/0022-1031(74)90070-5

[B17] Féry C. (1993). German Intonational Patterns. Tübingen: Niemeyer.

[B18] Ford C. E. Fox B. A. Thompson S. A. (1996). Practices in the construction of Turns: the “TCU” revisited. Pragmatics 6, 427–454. 10.1075/prag.6.3.07for

[B19] Ford C. E. Thompson S. A. (1996). Interactional units in conversation: syntactic, intonational, and pragmatic recources for the management of turns, in Interaction and Grammar, eds Ochs E. Schegloff E. A. Thompson S. A. (Cambridge: Cambridge University Press), 134–184.

[B20] Fox J. Weisberg S. (2011). An R Companion to Applied Regression, 2nd Edn. Thousand Oaks, CA: SAGE Publications.

[B21] Gilles P. (2005). Regionale Prosodie im Deutschen. Variabilität in der Intonation von Abschluss und Weiterweisung. Number 6 in Linguistik - Impulse und Tendenzen. Berlin: de Gruyter.

[B22] Griffin Z. M. (2001). Gaze durations during speech reflect word selection and phonological encoding. Cognition 82, B1–B14. 10.1016/S0010-0277(01)00138-X 11672707 PMC5130081

[B23] Griffin Z. M. Bock K. (2000). What the eyes say about speaking. Psychol. Sci. 11, 274–279. 10.1111/1467-9280.00255 11273384 PMC5536117

[B24] Halekoh U. Højsgaard S. (2014). A kenward-roger approximation and parametric bootstrap methods for tests in linear mixed models the R package pbkrtest. J. Stat. Softw. 59, 1–30. 10.18637/jss.v059.i0926917999

[B25] Heldner M. Edlund J. (2010). Pauses, gaps and overlaps in conversations. J. Phonet. 38, 555–568. 10.1016/j.wocn.2010.08.002

[B26] Hjalmarsson A. (2011). The additive effect of turn-taking cues in human and synthetic voice. Speech Commun. 53, 23–35. 10.1016/j.specom.2010.08.003

[B27] Huettig F. Rommers J. Meyer A. S. (2011). Using the visual world paradigm to study language processing: a review and critical evaluation. Acta Psychol. 137, 151–171. 10.1016/j.actpsy.2010.11.003 21288498

[B28] Just M. A. Carpenter P. A. (1980). A theory of reading: from eye fixations to comprehension. Psychol. Rev. 87, 329–354. 10.1037/0033-295X.87.4.329 7413885

[B29] Kendon A. (1967). Some functions of gaze-direction in social interaction. Acta Psychol. 26, 22–63. 10.1016/0001-6918(67)90005-4 6043092

[B30] Kenward M. G. Roger J. H. (1997). Small sample inference for fixed effects from restricted maximum likelihood. Biometrics 53, 983–997. 10.2307/2533558 9333350

[B31] Lammertink I. Casillas M. Benders T. Post B. Fikkert P. (2015). Dutch and english toddlers use of linguistic cues in predicting upcoming turn transitions. Front. Psychol. 6:495. 10.3389/fpsyg.2015.00495 25964772 PMC4408756

[B32] Levinson S. C. (2012). Action formation and ascription, in The Handbook of Conversation Analysis, eds Sidnell J. Stivers T. (Chichester: John Wiley & Sons, Ltd.), 101–130.

[B33] Levinson S. C. (2016). Turn-taking in human communication origins and implications for language processing. Trends Cogn. Sci. 20, 6–14. 10.1016/j.tics.2015.10.010 26651245

[B34] Levinson S. C. Torreira F. (2015). Timing in turn-taking and its implications for processing models of language. Front. Psychol. 6:731. 10.3389/fpsyg.2015.00731 26124727 PMC4464110

[B35] Magyari L. de Ruiter J. P. Levinson S. C. (2017). Temporal preparation for speaking in question-answer sequences. Front. Psychol. 8:211. 10.3389/fpsyg.2017.00211 28270782 PMC5318421

[B36] Mirman D. (2014). Growth Curve Analysis and Visualization Using R. Chapman & Hall/CRC the R series. Boca Raton, FL: CRC Press/Taylor, Francis Group.

[B37] R Core Team (2014). R: A Language and Environment for Statistical Computing. Vienna: R Foundation for Statistical Computing.

[B38] Sacks H. Schegloff E. A. Jefferson G. (1974). A simplest systematics for the organization of turn-taking for conversation. Language 50, 696–735. 10.1353/lan.1974.0010

[B39] Schaffer D. (1983). The role of intonation as a cue to turn taking in conversation. J. Phonet. 11, 243–257.

[B40] Selting M. (2007). Lists as embedded structures and the prosody of list construction as an interactional resource. J. Pragmat. 39, 483–526. 10.1016/j.pragma.2006.07.008

[B41] Sjerps M. J. Meyer A. S. (2015). Variation in dual-task performance reveals late initiation of speech planning in turn-taking. Cognition 136, 304–324. 10.1016/j.cognition.2014.10.008 25522192

[B42] Stephens J. Beattie G. (1986). On judging the ends of speaker turns in conversation. J. Lang. Soc. Psychol. 5, 119–134. 10.1177/0261927X8652003

[B43] Stivers T. Enfield N. J. Brown P. Englert C. Hayashi M. Heinemann T.. (2009). Universals and cultural variation in turn-taking in conversation. Proc. Natl. Acad. Sci. U.S.A. 106, 10587–10592. 10.1073/pnas.0903616106 19553212 PMC2705608

[B44] Tanenhaus M. K. Magnuson J. S. Dahan D. Chambers C. (2000). Eye movements and lexical access in spoken-language comprehension: evaluating a linking hypothesis between fixations and linguistic processing. J. Psycholinguist. Res. 29, 557–580. 10.1023/A:1026464108329 11196063

[B45] von Essen O. (1956). Grundzüge der Hochdeutschen Satzintonation, 1st Edn. Düsseldorf: A. Henn.

[B46] Walker M. B. Trimboli C. (1984). The role of nonverbal signals in co-ordinating speaking turns. J. Lang. Soc. Psychol. 3, 257–272.

[B47] Wesseling W. Son R. J. J. H. V. (2005). Timing of experimentally elicited minimal responses as quantitative evidence for the use of intonation in projecting TRPs, in Proceedings of Interspeech 2005, Vol. 6, (Lisbon), 3389–3392.

